# Feasibility of Home Parenteral Nutrition in Patients with Intestinal Failure Due to Neuroendocrine Tumours: A Systematic Review

**DOI:** 10.3390/nu15173787

**Published:** 2023-08-30

**Authors:** Dominique S. V. M. Clement, Sarah E. Brown, Mani Naghibi, Sheldon C. Cooper, Margot E. T. Tesselaar, Monique E. van Leerdam, John K. Ramage, Rajaventhan Srirajaskanthan

**Affiliations:** 1 Kings Health Partners, ENETS Centre of Excellence, Institute of Liver Studies, King’s College Hospital, London SE5 9RS, UK; 2 Department of Gastroenterology, King’s College Hospital, London SE5 9RS, UK; 3 Intestinal Rehabilitation Unit, St Mark’s and Northwick Park Hospitals, London HA1 3UJ, UK; 4 Department of Gastroenterology, University Hospital Birmingham, Birmingham B75 7RR, UK; 5 Department of Gastrointestinal Oncology, Netherlands Cancer Institute, ENETS Centre of Excellence, 1066 CX Amsterdam, The Netherlands; 6 Department of Gastroenterology and Hepatology, Leiden University Medical Center, 2333 ZA Leiden, The Netherlands

**Keywords:** neuroendocrine tumour, short bowel syndrome, inoperable malignant bowel obstruction, home parenteral nutrition, small bowel, survival

## Abstract

Introduction: Maintaining adequate nutritional status can be a challenge for patients with small bowel neuroendocrine tumours (NETs). Surgical resection could result in short bowel syndrome (SBS), whilst without surgical resection there is a considerable risk of ischemia or developing an inoperable malignant bowel obstruction (IMBO). SBS or IMBO are forms of intestinal failure (IF) which might require treatment with home parenteral nutrition (HPN). Limited data exist regarding the use of HPN in patients with small bowel neuroendocrine tumours, and it is not frequently considered as a possible treatment. Methods: A systematic review was performed regarding patients with small bowel NETs and IF to report on overall survival and HPN-related complications and create awareness for this treatment. Results: Five articles regarding patients with small bowel NETs or a subgroup of patients with NETs could be identified, mainly case series with major concerns regarding bias. The studies included 60 patients (range 1–41). The overall survival time varied between 0.5 and 154 months on HPN. However, 58% of patients were alive 1 year after commencing HPN. The reported catheter-related bloodstream infection rate was 0.64–2 per 1000 catheter days. Conclusion: This systematic review demonstrates the feasibility of the use of HPN in patients with NETs and IF in expert centres with a reasonable 1-year survival rate and low complication rate. Further research is necessary to compare patients with NETs and IF with and without HPN and the effect of HPN on their quality of life.

## 1. Introduction

Neuroendocrine neoplasms are rare neoplasms arising anywhere in the body that are mainly located within the gastroenteropancreatic (GEP) tract [1]. Neuroendocrine neoplasms are divided into two groups: neuroendocrine tumours (NETs) and neuroendocrine carcinomas (NECs) [2]. NETs differ from NECs based on their morphology and prognosis [3]. Home parenteral nutrition (HPN) would normally be indicated in cancers with a better prognosis [4]; hence, this systematic review focuses on patients with NETs only.

Within the GEP tract, the small bowel is one of the main sites of primary tumour [5]. Small bowel NETs are usually small in size and often metastasize to the mesenteric lymph nodes [6]. Within these mesenteric lymph nodes, they cause the fixation and calcification of the mesentery due to the production of hormones and growth factors [7,8,9]. This mesenteric lymph node mass can cause symptoms of bowel obstruction or small bowel ischemia. The preferred treatment is a resection of the small bowel primary with the mesenteric lymph node mass; however, this is not always possible due to its close relationship with the superior mesenteric artery (SMA) or superior mesenteric vein (SMV) [10,11,12]. If resection cannot be performed, the patient is at risk of developing an inoperable malignant bowel obstruction (IMBO). If resection is attempted, large segments of the small bowel would be resected, resulting in short bowel syndrome (SBS). IMBO and SBS are forms of intestinal failure (IF) where HPN can be helpful to maintain nutritional status. The use of HPN facilitates bowel rest, which results in fewer episodes of bowel obstruction or ischemia and improved quality of life. In patients with maintained nutritional status due to HPN, systemic anti-cancer treatments could be offered [13,14,15]. 

Home parenteral nutrition is a form of medical nutrition therapy, providing intravenous nutrition through a central venous catheter. Long-term HPN is usually provided in the home situation at night and managed by specialised centres [4]. One of the main life-threatening complications is sepsis due to catheter-related bloodstream infections (CRBSI). Other complications such as central venous thrombosis, catheter obstruction, and HPN-related liver disease have been reported [4]. 

The current view regarding surgical approaches for small bowel NETs is to avoid short bowel syndrome and subsequently the use of HPN. Limited literature exists to support this opinion [6,10,11,12]. In the case of an inoperable malignant bowel obstruction, no literature exists on how to deal with this in patients with NETs. The European Society of Parenteral and Enteral Nutrition (ESPEN) guidelines recommend HPN to prevent early mortality from malnutrition in patients with advanced cancer and intestinal failure (IF) if their life expectancy in relation to the cancer is expected to be longer than 1–3 months [4]. However, this guideline is mainly based on studies in patients with gastrointestinal, gynaecological, or head and neck cancers and does not include studies regarding patients with NETs. In general, patients with NETs have a better prognosis compared to the aforementioned cancers and, even in metastatic cases, the prognosis can be 5–7 years [16,17]. Despite these prognostic differences and current guidelines, HPN is not considered as a treatment due to poor awareness and concerns regarding feasibility and complications. 

Therefore, we performed a systematic review to summarize the currently available literature regarding the feasibility of the use of HPN in patients with intestinal failure due to NETs, including survival times and complication rates.

## 2. Materials and Methods

This systematic review was performed based on the Cochrane Handbook for Systematic Reviews and Interventions, version 6.2, 2021 [18] and the PRISMA 2020 guidelines [19]. 

### 2.1. Literature Search

A systematic search of MEDLINE, Embase, and CINAHL was conducted on 3 March 2023. There was no period limitation for the databases. The search string for each database is shown in Appendix A. Congress abstracts from the most relevant organisations are published in peer-reviewed journals and captured within the database search, and therefore a separate search for congress abstracts was not performed. 

Inclusion criteria were any studies reporting the use of HPN in adults (>18 years) with neuroendocrine tumours and intestinal failure published in the English language. 

Exclusion criteria were studies regarding children, animals, other conditions causing intestinal failure, and any studies published in languages other than English. 

After screening titles and abstracts, a cross-referencing of eligible studies was performed to avoid missing relevant studies. 

Two authors (DC and SB) worked independently to select eligible studies. If there were discrepancies between the two authors, a third author (RS) was consulted for a final decision. 

### 2.2. Study Selection

Eligible studies were randomised controlled trials, case series or case reports of any size, and studies regarding HPN in patients with cancer which included a subgroup of any number of patients with NETs. The studies should have reported on the use of HPN, survival of the patients on HPN, or the duration of HPN. Studies should also have reported on HPN-related complications. If studies reported on survival or complications only, they were also included. Studies that included a subgroup of patients with NETs as part of a larger study on patients with intestinal failure due to any form of cancer and reporting on survival and or complications were also included. 

### 2.3. Data Extraction and Synthesis

From the included studies, two authors (DC and SB) extracted the author’s name, the year of publication, the study country, study period, and study design, the sample size, gender, median age, subtypes of intestinal failure (SBS, IMBO, fistula), period on HPN, survival on HPN, catheter-related bloodstream infection rate, other complications (central venous thrombosis and HPN-related liver disease), and quality of life data, and presented the results in narrative result tables (1–3). The period on HPN and median survival data were extracted from the studies and converted into the number of patients on HPN longer than 1, 2, and 3 years. The number of catheter-related bloodstream infection rates was extracted from the studies and converted into the number per 1000 catheter days. Numbers of central venous thrombosis and HPN-related liver disease were also extracted from studies and presented as percentages.

### 2.4. Quality Assessment

The quality of studies, publication bias, and heterogeneity were assessed using the Cochrane risk of bias assessment tool (RoB2) [20].

## 3. Results

### 3.1. Literature Search

**Figure 1:**
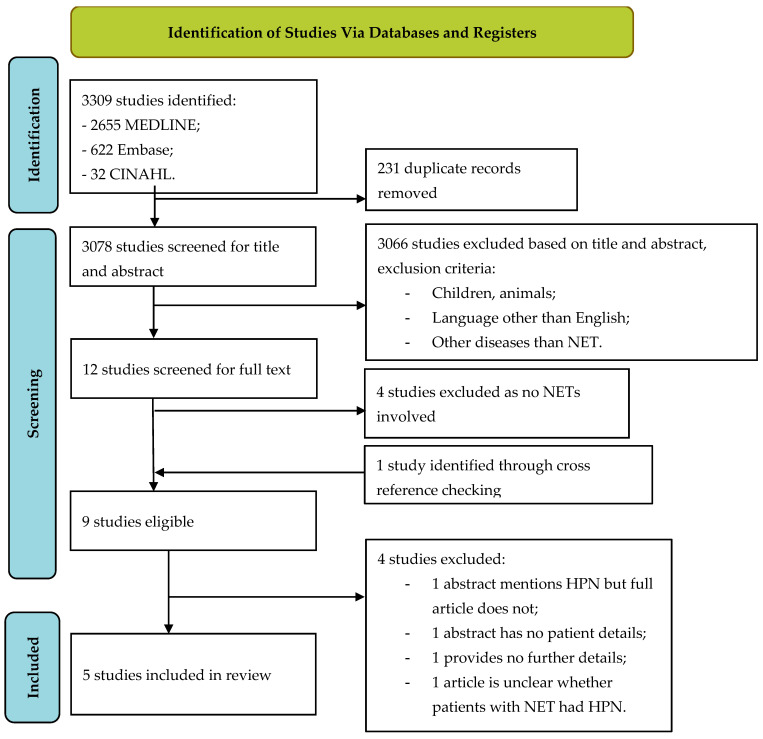
Flow chart for study selection NET neuroendocrine tumours with HPN home parenteral nutrition.

Searches in MEDLINE, Embase, and CINAHL resulted in 2655, 622, and 32 possible studies, respectively. After removing duplicates, 3078 studies remained. After screening titles and abstracts for exclusion criteria, nine studies were eventually included. From the nine articles, four were further excluded. There were two studies regarding the same patient from the same group of authors (one abstract and one full text article); the abstract mentions the use of HPN in one patient, while the full article does not mention this. We contacted the authors to clarify this but did not receive an answer. The abstract provided too few details regarding the patient with NETs on HPN for further analysis. One full text article mentions one patient with an NET on HPN but not with enough data to include in the analysis. The corresponding author was not able to provide the missing data. One article regarding inoperable malignant bowel obstructions mentions NETs as the cause for obstructions in two patients, but details regarding HPN use could not be extracted from the article and the authors did not respond to emails requesting additional information. This resulted in five articles for inclusion in this systematic review. The selection process is displayed in the flow chart in Figure 1.

### 3.2. Study Selection

Between 2001 and 2021, there were five studies published regarding intestinal failure including patients with NETs, three studies that reported on patients with NETs only, and in two studies, patients with NETs were a subgroup. Table 1 summarizes the details of all studies. There were no studies comparing patients with NETs and HPN and without HPN. 

In total, 65 patients with intestinal failure and the use of HPN could be identified and data from 60 patients was extracted from the studies for analysis. One article included 10 patients with NETs as part of a larger study cohort regarding the use of HPN in patients with advanced cancer. From five patients with NETs, details could be extracted for this review. No details from the remaining five patients were available. Four out of five studies reported on the use of HPN in patients with NETs. One study examined the effect of long-acting octreotide on patients with short bowel syndrome and included one patient with an NET who was on HPN for 10 years prior to enrolling in the study.

### 3.3. Feasibility of HPN

Table 1 summarizes the details of the five included studies and the baseline details of the patients. A total of 60 patients were included, 26 (43%) males and 34 females (56%), and the median reported age was 63–72 years. In total, 37 (62%) patients received HPN for short bowel syndrome, 21 (35%) patients for an inoperable malignant bowel obstruction, and 2 patients (3%) for a fistula.

**Figure 2:**
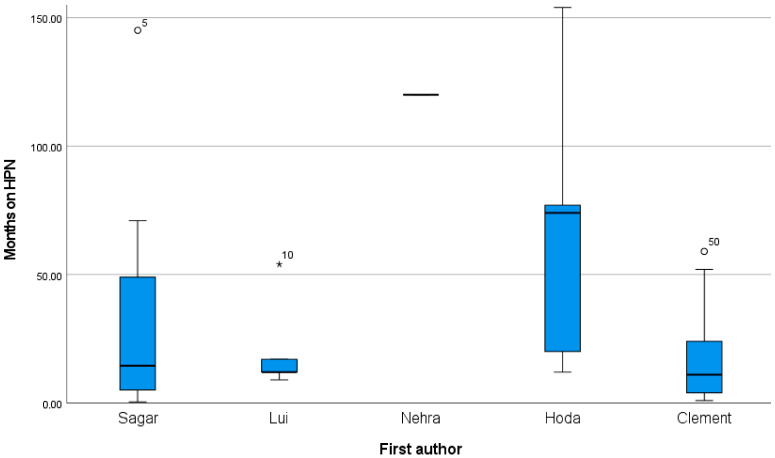
Box-plot overall survival. * outliers.

The overall survival time varied between 0.5 and 154 months (twelve years and 10 months) in the reported studies, as demonstrated in Figure 2 and Table 2. Not all studies provided details regarding the period on HPN and survival time. Fifty-eight percent (58%) of patients were still on HPN after 1 year, reducing to 32% after 2 years, and 22% after 3 years. 

Only the study from Clement et al. [21] compared patients with SBS and IMBO and found a significant difference in overall survival on HPN, with a median of 24 months for SBS versus 7 months for IMBO with a *p*-value 0.0009. The numbers of patients in the other studies were too small to explore the overall survival differences between SBS and IMBO.

### 3.4. Complications of HPN

The main complication of HPN, catheter-related bloodstream infection rate, is reported in four out of five studies. Other complications such as central venous thrombosis and HPN-related liver disease are reported in two studies only.

Three articles included only patients with NETs, and the two other articles included a subgroup of patients with NETs. One of those studies reported on HPN-related complications for the entire study cohort, not specifically for the patients with NETs. Since the patients with NETs are part of these studies, it can be assumed that the complication risks are applicable for patients with NETs. The other study does not mention any HPN-related complications. The results are summarized in Table 3. 

### 3.5. Quality Assessment

All studies included some major concerns regarding bias and none of the studies were free of possible bias. Table 4 summarizes the bias assessment. The studies from Sagar et al. and Nehra et al. received research funding grants from Novartis, the study from Lui et al. received a research grant from the Chinese Postdoctoral Science Foundation, and the study from Nehra et al. received funding from the National Institutes of Health, while the other two studies did not receive any funding. 

## 4. Discussion

This systematic review identified five studies, including 60 patients, to demonstrate the feasibility of using home parenteral nutrition in patients with NETs and intestinal failure. Interestingly, in these highly selected cases, 58% of these patients were still alive 1 year after commencing HPN. The complication rate was low, with a catheter-related bloodstream infection rate of only 0.64–2 per 1000 catheter days. 

This review contributes towards evidence regarding the feasibility of the use of HPN in patients with NETs and intestinal failure. The current view within the surgical neuroendocrine tumours community is to avoid surgical resection in case there is a risk of short bowel syndrome [6,10,11,12]. The view of SBS compromising on patient’s quality of life is supported by limited evidence [6,10,11,12]. Unfortunately, only one of the studies in this review included quality-of-life data, and this was regarding one patient prior to commencing HPN and one patient after commencing HPN [22]. Patients on HPN without cancer report conflicting satisfaction with their situation. In studies based on interviews, these patients report an improvement in quality of life since starting HPN and a good overall quality of life [26,27,28], while other studies based on questionnaires report on impaired quality of life [29,30,31]. In patients with advanced cancer who commence HPN, there is an improvement in quality of life, as HPN covers all nutritional needs, patients feel more secure that they are meeting their nutritional needs, the anxiety and distress regarding eating or weight loss reduces, and studies demonstrated a positive effect on well-being [32,33,34,35,36,37]. The current ESPEN Guidelines recommend HPN to prevent early mortality from malnutrition in patients with advanced cancer and intestinal failure (IF) if their life expectancy related to the cancer is expected to be longer than 1–3 months [4]. Nearly 60% of patients with NETs and intestinal failure on HPN in this review were alive 1 year after starting HPN, which would point towards more eligible patients with NETs for HPN treatment. Compared to studies regarding patients with intestinal failure due to non-cancer-related causes, the 1 year survival rate is 86–93% [38,39,40]. The difference in survival could be explained by comparing patients with NETs to patients without cancer. In patients with NETs, survival is related to the grade and stage of the disease, but even in metastatic cases, the 5-year survival rate is 70% [16,41]. The most relevant comparison is with non-NET cancer patients with IF treated with HPN, which was demonstrated in a recent systematic review and meta-analysis as a median survival of 7 months [42], hence the survival of NET patients with IF treated with HPN is favorable. The findings regarding 1 year survival in our review are in line with a study by Noelting et al., regarding a subgroup of 22 patients with SBS due to gynaecological or gastrointestinal cancers who had a median overall survival time of 30 months [43]. In the case of inoperable malignant bowel obstructions in patients with non-NET cancers, the median survival time was 3–4 months [44,45]. The difference with the findings in the current review could be explained by the difference in nature of NETs compared to the non-NET cancers in the aforementioned studies. 

The studies in the current review showed a catheter-related bloodstream infection rate of 0.5–2 per 1000 catheter days. This is in line with other studies regarding CRBSI in patients without cancer (0.33–1.44 per 1000 catheter days) and CRBSI in patients with cancer (0.27–2.78 per 1000 catheter days) [42,46,47]. The findings from this review are also in line with the recommendations from the ESPEN Guidelines, where a CRBSI rate of 0.4–3.0 per 1000 catheter days is acceptable [4]. Not all studies in this systematic review reported on other complications from HPN. We identified five cases of central venous thrombosis, which is in line with the findings of other studies regarding HPN in patients with cancer which reported an incidence rate of 0.09–4.34 per 1000 catheter days [40,47,48]. In this systematic review, we recorded three episodes of HPN-related liver disease, and in the literature a rate of 8% in patients with cancer on HPN is reported [40]. 

Despite an extensive search in the most common databases, Medline, Embase, and CINAHL, and the use of broad search terms, we could only identify five eligible studies including 60 patients with NETs and intestinal failure and the use of HPN. This suggests there is a form of bias, only reporting patients with effective treatment of HPN for intestinal failure, or a generalised under-utilisation of HPN for the NET patient population with IF. This may be because NETs are mainly managed in expert centres and intestinal failure is managed by separate specialised centres, which could result in inadequately identifying patients eligible for HPN treatment [4]. There is a bias, as two studies reported having identified more patients with NETs and IF and HPN but could not report on details [21,24]. Another concern regarding bias is the lack of a comparison group of patients with NETs and intestinal failure without HPN. It is unethical to randomize patients with intestinal failure between HPN and standard care; patients on standard care are deprived from nutrition and likely to die from starvation. However, case matching with patients who have not received HPN could be possible. 

There is also a risk of bias in reporting catheter-related bloodstream infection. Since HPN is usually organised by expert regional hospitals, those hospitals could miss some complications as they might be dealt with in local hospitals without informing the HPN expert centre. 

This review included five studies regarding patients with NETs, IF, and the use of HPN. The strength of this review is the extensive search, summary of available data, and comparison with research regarding HPN in non-NET cancers. There are concerns regarding the bias of the included studies. As the included studies are case series only, no meta-analysis could be performed. 

Future research should focus on the comparison of patients with NETs and IF with and without HPN regarding quality of life, survival time, and complications. A higher level of knowledge of the availability and outcome of HPN treatment for patients with IF caused by NET and closer collaboration between NET and HPN centres would lead to improved patient identification for this treatment.

## 5. Conclusions

This review demonstrates the feasibility of the use of HPN in patients with NETs and IF in highly specialised centres, with a favourable 1-year survival rate and a low complication rate. The use of HPN for non-NET cancer patients with IF is widely established. Despite the longer survival demonstrated in this study for NET patients with IF treated with HPN, there appears to be an under-utilisation of HPN in this group. The results of this study could support clinicians in the NET community to consider HPN more readily when IF occurs and guide their discussions with patients with NETs at risk of developing intestinal failure. 

## Figures and Tables

**Table 1:** Study results.

Author, Year	Country	Study Period	Study Design	Sample Size	Gender	Median Age (Year)	Intestinal Failure Subtypes	Outcomes
Clement D 2023 [21]	The Netherlands and the United Kingdom	2000–2019	Retrospective case series	*n* = 41	Male *n* = 18 (44%) Female *n* = 23 (56%)	65	SBS *n* = 27 (66%) IMBO *n* = 14 (34%)	Survival, catheter-related bloodstream infection rate
Sagar V 2020 [22]	United Kingdom	2000–2017	Retrospective case series	*n* = 8	Male *n* = 5 (63%) Female *n* = 3 (37%)	NR	SBS *n* = 4 (50%) IMBO *n* = 2 (25%) Fistula *n* = 2 (25%)	Survival, catheter-related bloodstream infection rate, quality of life
Liu M 2020 [23]	United Kingdom	2010–2019	Retrospective case series	*n* = 5	Male *n* = 2 (40%) Female *n* = 3 (60%)	63	SBS *n* = 2 (40%) IMBO *n* = 3 (60%)	Period on HPN, survival, HPN-related complications
Hoda D 2005 [24]	United States	1979–1999	Retrospective case series	*n* = 5 **	Male *n* = 1 (20%) Female *n* = 4 (80%)	64	SBS *n* = 3 (60%) IMBO *n* = 2 (40%)	Survival and HPN-related complications
Nehra V 2001 [25]	United States	NR	Prospective case series (one arm)	*n* = 1 ***	Female *n* = 1 (100%)	72	SBS *n* = 1	Body weight, stool fat, sodium and potassium, gastric- and small bowel transit times

NR: not reported, SBS: short bowel syndrome, IMBO: inoperable malignant bowel obstruction, HPN: home parenteral nutrition; ** study included 52 patients, 10 patients with NETs, and for 5 patients only details are available; *** study included 8 patients, 1 with NETs.

**Table 2:** Summary period on HPN.

	Number of Patients with NETs	Median Survival (Months on HPN)	Median Survival (Months) Short Bowel Syndrome	Median Survival (Months) Inoperable Malignant Bowel Obstruction	Number of Patients on HPN Longer than 1 Year	Number of Patients on HPN Longer than 2 Years	Number of Patients on HPN Longer than 3 Years
Clement D 2023 [21]	*n* = 41	19 months (IQR 7–50)	24 months (IQR 12–52)	7 months (IQR 3–19)	*n* = 20	*n* = 11	*n* = 6
Sagar V 2020 [22]	*n* = 8	27 months (IQR 0–54)	NR	NR	*n* = 5	*n* = 3	*n* = 2
Liu M 2020 [23]	*n* = 5	12 months (IQR 9–54)	35.5 months (no IQR)	12 months (no IQR)	*n* = 4	*n* = 1	*n* = 1
Hoda D 2005 [24]	*n* = 5	74 months (IQR 16–115)	74 months (no IQR)	44.5 months (no IQR)	*n* = 5	*n* = 3	*n* = 3
Nehra V 2001 [25]	*n* = 1	120 months	120 months	0	*n* = 1	*n* = 1	*n* = 1
Total	*n* = 60				*n* = 35 (58%)	*n* = 19 (32%)	*n* = 13 (22%)

NR not reported, IQR interquartile range.

**Table 3:** HPN-related complications.

	Number of Patients with NETs	Number of Catheter-Related Bloodstream Infections Reported	Catheter-Related Bloodstream Infection Rate/1000 Catheter Days	Central Venous Thrombosis	HPN-Related Liver Disease
Clement D 2023 [21]	*n* = 41	*n* = 23	1/1000 catheter days	NR	NR
Sagar V 2020 [22]	*n* = 8	*n* = 4	2/1000 catheter days	NR	NR
Liu M 2020 [23]	*n* = 5	*n* = 2	0.64/1000 catheter days	*n* = 1 (20%)	*n* = 1 (20%)
Hoda D 2005 ** [24]	*n* = 5	*n* = 18	0.97/1000 catheter days	*n* = 4 (8%)	*n* = 2 (4%)
Nehra V 2001 [25]	*n* = 1	NR	NR	NR	NR

NR: not reported, ** based on entire study cohort (*n* = 52 patients).

**Table 4:** Bias assessment.

	Randomisation Process (D1)	Deviations from the Intended Interventions (D2)	Missing Outcome Data (D3)	Measurement of the Outcome (D4)	Selection of the Reported Result (D5)	Overall
Clement D 2023 [21]	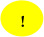	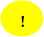	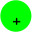	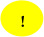	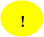	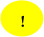
Sagar V 2020 [22]	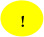	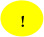	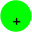	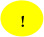	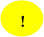	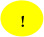
Liu M 2020 [23]	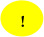	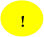	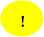	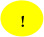	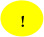	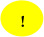
Hoda D 2005 [24]	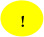	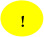	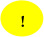	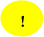	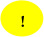	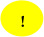
Nehra V 2001 [25]	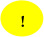	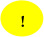	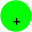	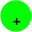	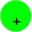	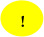

+ low risk of bias, ! unclear risk of bias.

## Data Availability

All data used in this systematic review are extracted from original publications.

## Appendix A. Search String

### Search Strategy

**MEDLINE**

#1 (carcinoma, neuroendocrine [MeSH Terms]) OR (neuroendocrine tumor [MeSH Terms]) OR (neuroendocrine tumors [MeSH Terms]) OR (carcinoid [MeSH Terms])

#2 (neuroendocrine * [Title/Abstract]) OR (carcinoid [Title/Abstract]) OR (endocrine * [Title/Abstract])

#3 #1 or #2

#4 (parenteral * [Title/Abstract]) OR (nutrition [Title/Abstract]) OR PN [Title/Abstract]) OR (HPN [Title/Abstract]) OR (TPN [Title/Abstract]) OR (intestinal failure [Title/Abstract])

#5 (feeding, parenteral [MeSH Terms]) OR (feeding, home parenteral [MeSH Terms]) OR (total parenteral nutrition, home [MeSH Terms]) OR (total parenteral nutrition [MeSH Terms]) OR (nutrition, total parenteral [MeSH Terms]) OR (nutrition, home total parenteral [MeSH Terms] OR (home total parenteral nutrition [MeSH Terms])

#6 #4 OR #5

#7 #3 AND #6

**Embase**

#1 neuroendocrine tumor/or endocrine tumor/or carcinoid/or gastroenteropancreatic neuroendocrine tumor/or neuroendocrine cell line

#2 neuroendocrine *.mp.

#3 carcinoid.mp.

#4 #2 or #3

#5 #1 OR #4

#6 exp short bowel syndrome/or exp intestinal failure/or exp parenteral nutrition/or exp intestine absorption

#7 malnutrition

#8 parenteral nutrition.mp.

#9 intestinal failure.mp.

#10 short bowel syndrome.mp

#11 #6 OR #7

#12 #11 OR #8

#13 #12 OR #9

#14 #13 OR #10

**CINAHL**

#1 (MH “Neuroendocrine Tumors”) OR (MH “Carcinoma, Neuroendocrine”) OR (MH “Carcinoid Tumor”) OR “neuroendocrine tumors OR neuroendocrine carcinoma OR neuroendocrine cancer OR carcinoid tumor OR carcinoid”

#2 TX neuroendocrine tumors OR TX neuroendocrine cancer OR TX neuroendocrine carcinoma OR TX carcinoid tumor OR TX carcinoid

#3 #1 OR #2

#4 (parenteral nutrition or total parenteral nutrition or tpn) OR intestinal failure OR short bowel syndrome OR small bowel obstruction

#5 TX (parenteral nutrition or total parenteral nutrition or tpn) TX intestinal failure OR TX short bowel syndrome OR TX small bowel obstruction

#6 #4 and #5

#7 #3 AND #6
